# Domestic donkey bite of genitalia: an unusual etiology of penile glans amputation in Burkina Faso (case report and literature review)

**DOI:** 10.11604/pamj.2020.36.13.20620

**Published:** 2020-05-12

**Authors:** Adama Ouattara, Clotaire Yaméogo, Abdoul Karim Paré, Aristide Fasnéwindé Kaboré, Désiré Ky, Boukary Kabré, Amidou Bako, Delphine Yé, Timothée Kambou

**Affiliations:** 1 Division of Urology, Souro Sanou University Teaching Hospital, Bobo-Dioulasso, Burkina Faso; 2 Division of Urology, Yalgado Ouedraogo University Teaching Hospital, Ouagadougou, Burkina Faso

**Keywords:** Donkey bite, penile glans, amputation, external genitalia

## Abstract

A 15-years-old boy was admitted in our hospital emergency unit with complaints of injured and hemorrhagic penile glans after donkey bites. The accident occurred during domestic activity with the animal when the boy attempted to guide the donkey. After thorough physical examination, the patient presented a penile glans amputation. Tetanic prophylaxis was started. The patient benefited from hemostatic suturing, stump regularization and penile glans reconstructive surgery and there was no complication. Functional and cosmetic results were satisfactory with good quality of micturition after six months’ follow-up.

## Introduction

Bite wounds are relatively frequent, the order of frequency being, dogs, cats and humans [1]. The mammalian bites are reported to account for 1% of all visits to emergency departments [2]. Apart from dog and cat, other animals such as cow, monkey, horse, pig, camel are reported responsible for bite injuries [1,2]. In spite of the large variety of bite injuries and their localization on the human body, donkey bites are not common and rarely affect the genital region [3]. Herein, we present a rare case of partial penile amputation as a result of a domestic donkey bite in a rural young boy.

## Patient and observation

**Figure 1:**
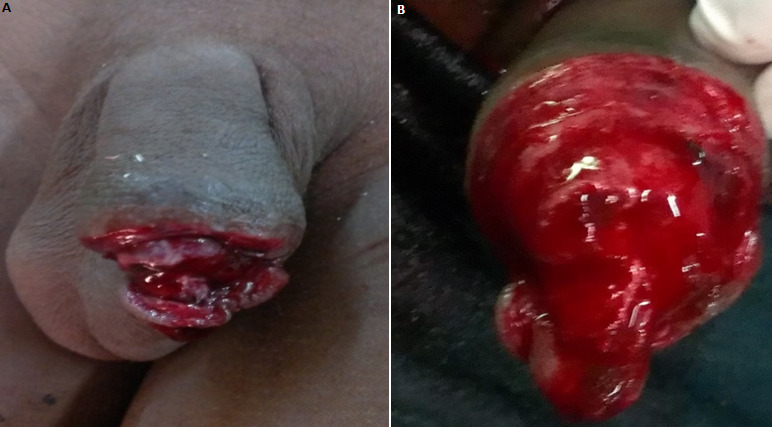
Hemorrhagic wound of the distal part of the penis with partial amputation of two thirds of the penile glans

**Figure 2:**
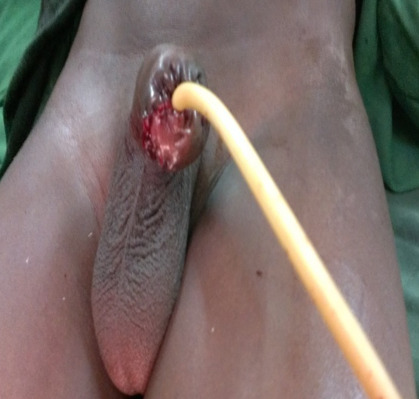
Hemostasis and regularization of the remaining penile stump and partial glandular reconstruction with a CH 12 urethral catheter

**Figure 3:**
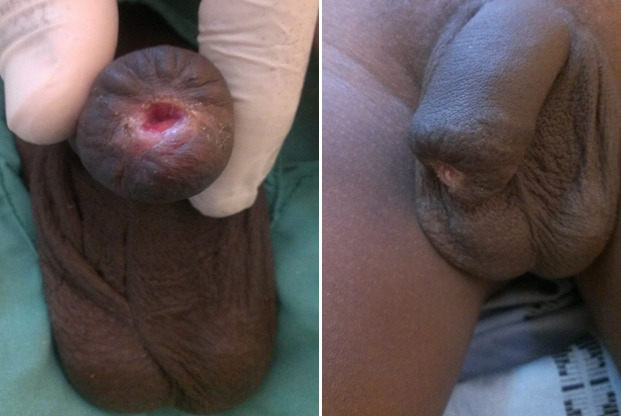
Acceptable penile cosmetic appearance

A 15-year-old boy, living in a rural area, no longer attending school, was referred in February 2016 by a primary health center to the emergency unit of the University Hospital of Souro Sanou, for external genitalia injury after a donkey bite. The bite would have occurred when the boy was guiding the animal pulling a cart as part of the household activities. The anamnesis did not reveal any particular pathological history and his vaccination status regarding to tetanus and rabies vaccines was unknown. After emergency treatment with dressing and tetanus prophylaxis at the peripheral health center, the child was received at the 5^th^ hour in our hospital emergency unit. At the time of admission, thorough clinical examination revealed that the patient was in good general condition, conscious and apyretic. He had a hemorrhagic wound of the distal part of the penis with partial amputation of two thirds of the penile glans; the perineal region, scrotum and testicles were intact (Figure 1). Emergency admission to the operating room after preoperative preparation, surgical exploration under general anaesthesia of the penile bite revealed a loss of the penile glans, the glandular urethra with exposure of the cavernous and spongious bodies of the distal penis. After thorough washout of the wounds with saline solution, and careful debridement, we proceeded to control the bleeding by performing careful hemostasis, regularization of the remaining penile stump and partial glandular reconstruction with a CH 12 urethral catheter held in place for 3 weeks (Figure 2). As the patient had already received a tetanus vaccine in the primary center, we carried out tetanus serotherapy, followed by a broad-spectrum antibiotic prophylaxis based on cephalosporin and imidazole. The rabies vaccination was not carried out after evaluation and the animal was submitted to surveillance. Functional and cosmetic results at three and six months of follow-up were acceptable with good urinary function, and an acceptable penile cosmetic appearance (Figure 3). The functional result regarding sexual function was not evaluated with the residual penile stump due to the patient's young age.

## Discussion

Animal bites are relatively common involving various animal species. Bites caused by cattle, donkeys and camels have been described in the literature [1-3]. Similarly, the locations of these bites are as diverse as they are varied, but bites on the external genitalia are rare [3]. Over a 15-year period, Jaindl M *et al.* recorded more than 5000 cases of bite injuries in children, young adults and adults and found that only seven cases were genital lesions due to animal bites [4]. De Luca *et al.* found only 57 cases of male genitalia due to animal bites published worldwide [3]. The donkey scientifically known as *Equus africanus asinus* is a domestic animal involved in farm and domestic work in many countries in sub-Saharan Africa. Thus donkey-drawn carts are widely used in our context and most accidents involving them occur in the context of rural or domestic work. Unlike other pets, donkeys are not often responsible for human injuries compared to other animals such as dogs and cats. However, when they occur, these donkey bites are sometimes dramatic and fatal [5]. Of all donkey-related injuries to the human body, the external genitalia are rarely affected [3]. In fact, penile lesions due to a domestic donkey bite are rare, perhaps because of the hidden aspect of genitalia [3,6].

Male genitalia have a huge capacity to resist injury. The flaccidity of the pendulum part of the penis limits the transfer of kinetic energy during a trauma. The bite of the donkey is responsible for penetrating wounds to the penis. Depending on the segment affected, penetrating lesions are generally associated with penile, testicular and/or pelvic lesions [7]. In our case, the glandular urethra was affected, this required the installation of a permanent urinary catheter for 3 weeks. The proper management of animal bites requires the prevention of tetanus, rabies and wound infection. Otherwise, the purpose of initial management of the animal bite is to free the patient from life-threatening complications such as sepsis, tetanus and, possibly, rabies. Indeed, wound bites carry a risk of infection, which often occurs within the first 48 hours after the injury [8]. Infectious organisms most often come from the mouth of the bite, but can also come from the host's flora or environment. Otherwise, the genital area of the body is considered a high-risk area for infection [8]. Despite the Cochrane published in 2012 [9] which concluded that prophylactic antibiotics did not appear to reduce the rate of infection after mammalian bites, unless hand bites, current guidelines still recommended antibiotic prophylaxis [10]. Antibiotic selection is generally based on the animal concerned. However, no specific recommendations mention the bite of the donkey. Our patient received antibiotics within 10 days based on 3G cephalosporin and imidazole. The prevention of tetanus has also been indicated and prescribed, regarding the unknown vaccination status of our patient. Otherwise, rabies vaccination is currently recommended for some cases, namely the elderly and immunocompromised patients. Penile amputation surgery is difficult because it has three objectives: urinary and sexual function and also a good aesthetic appearance. All cases of penile lesions by donkey bites reported in the literature successfully achieve these objectives. In our case, the partial reconstruction of the penis was performed in an emergency situation. Sexual activity with the residual penis may be difficult with the absence of a glans although this function has not been evaluated due to the young age of the child.

## Conclusion

Traumas and injury of external genitalia from donkey bites are not uncommon. They appear unusual but are often serious and can lead to compromise the functional and cosmetic prognosis of genitalia. The complications and sequelae can be very disabling with an unsatisfactory cosmetic appearance after surgical procedure. Management must be early in order to minimize the risk of disabling sequelae and psychological distress. Preventive measures must be observed through compliance with workplace safety rules to prevent the occurrence of such domestic accidents.

## Competing interests

The authors declare no competing interests.
